# Establishing family physician research networks in South Africa

**DOI:** 10.4102/safp.v62i1.5216

**Published:** 2020-11-05

**Authors:** Robert Mash

**Affiliations:** 1 Division of Family Medicine and Primary Care, Faculty of Medicine and Health Sciences, University of Stellenbosch, Cape Town, South Africa

**Keywords:** primary care research, family physicians, research networks, clinical practice research

## Abstract

Practice-based research networks have been an important strategy in many parts of the world to enable primary care research and involve clinicians. The Stellenbosch University Family Physician Research Network (SUFPREN) was established in 2017 to enable family physicians in clinical practice to engage with practical research that addressed key challenges and enhanced clinical governance. It is a collaboration between academic family physicians at the university and clinical family physicians in the district health services. In this article, the establishment of SUFPREN is described – membership of the network, selection of research questions, roles in the research process, communication and coordination, involvement of the Department of Health, types of questions and methods, data management, dissemination of findings and funding. The initial work of SUFPREN is described and a vision for future family physician research networks in South Africa is defined.

## Introduction

Practice-based research networks are a key feature of primary care research in many parts of the world, but are not yet established in South Africa.^1^ In this article, I describe the implementation of the Stellenbosch University Family Physician Research Network (SUFPREN) as an example of what is possible in our context and in the hope that other Departments of Family Medicine may emulate this model and eventually enable the creation of a South African Family Physician Research Network.

Family physicians are embedded in the district health services in primary care facilities and district hospitals. As such, they experience a wide range of clinical, health services and educational challenges on a daily basis. Many of these challenges raise questions that can be practically addressed through research. Indeed, many family physicians have ‘practical research’ as part of their job description and yet struggle to fulfil this aspect of their work. Research can be seen as part of the toolbox needed for clinical governance in order to improve the quality of service delivery and clinical outcomes.^2^ Despite the requirement to complete a research project and master the research process as part of their training, few family physicians in clinical practice continue to engage with research as clinician-scientists. The reasons for this are multiple and include lack of time, capability and confidence to initiate and conduct their own research.

Being part of a practice-based network with support from academic family physicians at the university can enable family physicians to participate in practical research. In addition, the network enables research to be conducted over multiple sites and geographical areas, which can increase the size, validity and generalisability of the work.

## Establishing Stellenbosch University Family Physician Research Network

The idea of SUFPREN grew out of the Division of Family Medicine and Primary Care at Stellenbosch University during our annual retreat and strategic planning in 2017. The concept was subsequently unpacked at a workshop with Prof. Felicity Goodyear-Smith (Chair of the World Organisation of Family Doctors (WONCA) Working Party on Primary Care Research), who shared some of the global experience.^3,4,5^ At this workshop, the family physicians defined their vision to improve district health services through the creation of new evidence that addresses community health needs, contributes to clinical governance and quality of services, and assists policy and decision makers in a resource constrained environment.

The mission was to establish a network of family physicians who can collaborate on practical research projects based on sub-district health needs. The network would be supported by academic family physicians and researchers based in the Division.

The additional potential benefits of the network were seen as stimulating research interest and capability amongst family physicians, improving job satisfaction and intellectual stimulation as well as enhancing institutional and personal academic status.

## Who is part of the network?

The network included two groups of people: academic family physicians or researchers based in the Division of Family Medicine and Primary Care at the university and family physicians located within the district health services and connected with Stellenbosch University.

Family physicians within the public sector of the Western Cape were invited to join the network. Altogether 25 family physicians joined the network from every district within the Western Cape. At the university three academic family physicians committed to support the network.

## How are research questions chosen?

One of the key principles was that research questions should emerge from clinical practice and be prioritised by members of the network. The academic family physicians needed to hold back from using the network to address their own questions and rather support clinicians to identify, prioritise and address their questions.

Family physicians brainstormed their research questions at the annual capacity building workshop facilitated by the Division. Research questions were collated, clarified and prioritised using the nominal group technique.

## Roles of researchers and clinicians

**FIGURE 1:**
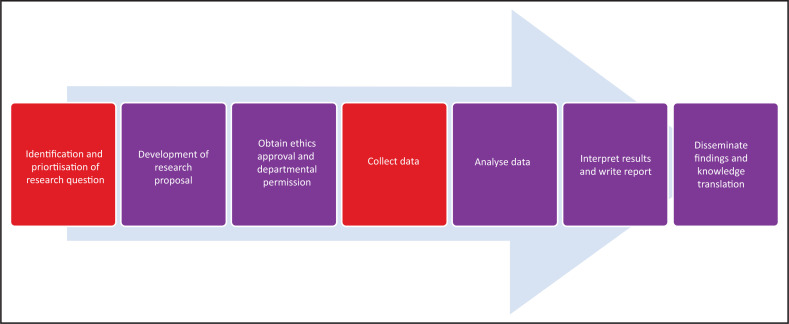
Steps in the research process.

The research process is illustrated in Figure 1 and the contribution of academic family physicians and clinicians is described in Table 1.

**TABLE 1:** Roles in research process.

Research steps	Role of academic family physicians/researchers	Role of clinical family physicians
Identification and prioritisation of research question	Facilitate process	Contribute ideas and prioritise
Development of research proposal	Conceptualise methods and write proposal	Provide feedback on feasibility of methods and validation of data collection tools
Obtain ethical approval and provincial permission	Apply for ethical approval and provincial permission	Assist with approval from provincial managers
Collect the data	Facilitate software or system of collecting and capturing data	Collect the data
Analyse the data	Analyse the data.	Provide feedback on results
Interpret results and write report	Draft the manuscript	Help interpret and discuss results and implications
Disseminate findings and knowledge translation	Support publication of the manuscript. Conference presentations and other media	Disseminate and use findings in the Department of Health

The two groups involved in the network took responsibility for different steps in this research process, although the whole process was co-ordinated by the university and each step would require collaboration from both the groups. The family physicians in clinical practice identified and prioritised the research questions, collected data, helped to interpret the results and edit the report as well as to disseminate and implement the findings. The academic family physicians or researchers on the other hand developed the research proposal and methodology, navigated ethical approval and obtained permission from the Department of Health, captured and analysed the data, drafted the report, submitted for publication and took the lead with dissemination. Although the academic family physicians conceptualised the study, the clinicians were important in validating and finalising the data collection tools, such as questionnaires or interview guides. Other researchers and statisticians can be engaged to assist with specific research projects or steps such as data analysis as necessary.

## Co-ordination, communication and meetings

One of the academic family physicians at the Division took responsibility for overall co-ordination of the network. Co-ordination involved communication (mostly email), monitoring progress and providing feedback to interested stakeholders. The co-ordinator was supported part-time by an administrative assistant. The network did not plan any additional face-to-face meetings, but used existing workshops and meetings to plan and communicate.

As the network gathered momentum, it became clear that multiple research projects could be addressed simultaneously as projects could be at different stages. For example, one project might be in analysis and report writing, another at data collection and another at conceptualisation and proposal writing. Although there was one coordinator for the network, different academics could take responsibility for leading different projects.

## Involvement of the Department of Health

At a provincial level, the network gave feedback to the Department of Health on research projects and disseminated results via publications, research days and conferences. At a district level, the family physicians engaged with key people (e.g. district manager, professional support services, programme managers, information managers) to share research questions, priorities, facilitate permission, access existing data or disseminate results. At a sub-district or facility level, the family physician engaged with the multidisciplinary clinical team and local managers to identify research questions, facilitate permission, collect data and disseminate results. The multidisciplinary team might include, for example, other family physicians or medical officers, clinical nurse practitioners, pharmacists, receptionists or community health workers.

## Types of research questions and methods

Whilst the specific research questions arose from the family physicians and their clinical practice teams, it is possible to conceptualise a broad range of questions that such a network could address using the following typology.^6^

- Basic research: research that develops the tools and methods needed.
- Clinical research: research that investigates specific diseases and conditions.
- Health services research: research that investigates cross-cutting issues in service delivery.
- Health systems research: research that investigates the system inputs and policy-related issues.
- Educational research: research that investigates training of the health workforce

Such a wide variety of questions can be addressed through the network by a variety of methods such as surveys, quality improvement cycles, randomised controlled trials, descriptive exploratory qualitative studies, programme evaluation and action research as well as mixed methods.^7^

## Data management

Data is collected by the family physician in their sub-district, sometimes with the help of other colleagues. Collection could be electronic or paper-based. The quality and integrity of data should be monitored by the project lead or network co-ordinator. Ideally data is collected electronically via the Internet or app using software such as RedCap. Data is then captured, and stored centrally and securely at the university. Data is checked and analysed by the academic family physicians using software such as the Statistical Package for Social Sciences or Atlas-ti. The other resources of the Faculty are available to assist, such as biostatistics.

## Dissemination of results

The findings should be published in a peer-reviewed scientific journal and the involvement of a network may raise questions of authorship. At this point, all family physicians involved directly in the study would meet the criteria for authorship as they would have selected the question, given feedback on the proposal, helped to validate the data collection tools, collected data and contributed to or provided feedback on the final manuscript.

As with all other research, dissemination should be planned via a variety of channels such as publications, conference presentations, social media, podcasts, issue- and policy-briefs. A knowledge translation strategy should be developed and followed.

## Funding

Research projects are not expected to require substantial funding as people will contribute as part of their job description and travel will be limited by the distributed nature of the network. However, some research might require additional funding that would be sought through the usual channels.

## Initial projects

The first project tackled by the network looked at the co-ordination of care between primary care and hospital out-patients.^8^ This study has been published and shared with the local department of health. As this study required data collection in primary care, it was difficult for those in district hospitals to contribute and highlighted a need to think more carefully about the location of research within the network in future studies.

The second project will look at the factors linked to the retention of medical officers in district health services. This project has been delayed by COVID-19, but at the same time, SUFPREN was able to quickly respond to the epidemic. One of the family physicians put forward a research question on the management of patients at district hospital level and due to the network, this question could be quickly developed and taken up. Currently eight district hospitals are collecting data.

## The way forward

It should be relatively easy to replicate such a network at other universities. Already, family physicians attached to the University of Cape Town are participating in the study on COVID-19 and district hospitals. If each Department of Family Medicine developed its own network, then this could not only facilitate an increase in relevant research at a local level but also build research capacity. There is also the potential to expand such networks into private practice.

In the longer term, it should be possible for networks attached to local universities to combine and work together on national research projects. The South African Academy of Family Physicians could potentially play a co-ordinating role for this at a national level.

## References

[1] Goodyear-Smith F, Mash B. International perspectives on primary care research. 1st ed. London: CRC Press, 2016; p. 255.

[2] Mash R, Blitz J, Malan Z, Von Pressentin K. Leadership and governance: Learning outcomes and competencies required of the family physician in the district health system. S Afr Fam Pract. 2016;58(6):232–235. 10.1080/20786190.2016.1148338

[3] Dolor RJ, Campbell-Voytal K, Daly J, et al. Practice-based Research Network Research Good Practices (PRGPs): Summary of recommendations. Clin Transl Sci. 2015;8(6):638–646. 10.1111/cts.12317 26296309 PMC5351126

[4] Campbell-Voytal K, Daly JM, Nagykaldi ZJ, et al. Team science approach to developing consensus on research good practices for practice-based research networks: A case study. Clin Transl Sci. 2015;8(6):632–637. 10.1111/cts.12363 26602516 PMC5351116

[5] Van Weel C, Rosser WW. Improving health care globally: A critical review of the necessity of family medicine research and recommendations to build research capacity. Ann Fam Med [serial online]. 2004 [cited 2020 Aug 17]. Available from: https://www.annfammed.org/content/2/suppl_2/S5.short 10.1370/afm.194 PMC1466768 15655089

[6] Beasley JW, Starfield B, Van Weel C, Rosser WW, Haq CL. Global health and primary care research. J Am Board Fam Med. 2007;20(6):518–526. 10.3122/jabfm.2007.06.070172 17954858

[7] Goodyear-Smith F, Mash B. How to do primary care research. 1st ed. London: CRC Press; 2018.

[8] Mash R, Steyn H, Bello M, et al. The quality of feedback from outpatient departments at referral hospitals to the primary care providers in the Western Cape: A descriptive survey. S Afr Fam Pract. 2019;61(6):252–259. 10.1080/20786190.2019.1676021

